# Impact of chemotherapy and immunotherapy on the composition and function of immune cells in COVID-19 convalescent with gynecological tumors

**DOI:** 10.18632/aging.203739

**Published:** 2021-12-04

**Authors:** Tianyu Qin, Ensong Guo, Funian Lu, Yu Fu, Si Liu, Rourou Xiao, Xue Wu, Chen Liu, Chao He, Zizhuo Wang, Xu Qin, Dianxing Hu, Lixin You, Fuxia Li, Xi Li, Xiaoyuan Huang, Ding Ma, Xiaoyan Xu, Bin Yang, Junpeng Fan

**Affiliations:** 1 Department of Obstetrics and Gynecology, Tongji Hospital, Tongji Medical College, Huazhong University of Science and Technology, Wuhan 430030, China; 2 Cancer Biology Research Center, Tongji Hospital, Tongji Medical College, Huazhong University of Science and Technology, Wuhan 430030, China; 3 Department of Gynecology, Foshan Women and Children’s Hospital Affiliated to Southern Medical University, Foshan 528000, China; 4 Department of Cell, Development and Cancer Biology, Oregon Health and Sciences University, Portland, OR 97201, USA

**Keywords:** COVID-19, tumor, chemotherapy, ICIs, single cell sequencing

## Abstract

Ongoing pandemic and potential resurgence of Coronavirus disease 2019 (COVID-19) has prompted urgent efforts to investigate the immunological memory of convalescent patients, especially in patients with active cancers. Here we performed single-cell RNA sequencing in peripheral blood samples of 3 healthy donors (HDs), 4 COVID-19 patients (Covs) and 4 COVID-19 patients with active gynecological tumor (TCs) pre- and post- anti-tumor treatment. All Covs patients had recovered from their acute infection. Interestingly, the molecular features of PBMCs in TCs are similar to that in Covs, suggesting that convalescent COVID-19 with gynecologic tumors do not have major immunological changes and may be protected against reinfection similar to COVID-19 patients without tumors. Moreover, the chemotherapy given to these patients mainly caused neutropenia, while having little effect on the proportion and functional phenotype of T and B cells, and T cell clonal expansion. Notably, anti-PD-L1 treatment massively increased cytotoxic scores of NK cells, and T cells, and facilitated clonal expansion of T cells in these patients. It is likely that T cells could protect patients from SARS-CoV-2 virus reinfection and anti-PD-L1 treatment can enhance the anti-viral activity of the T cells.

## INTRODUCTION

The unprecedented outbreak of COVID-19, a new coronavirus disease caused by the new severe acute respiratory syndrome coronavirus-2, namely SARS-CoV-2, has led to more than 80 million infections and over 1,831,412 deaths as of Jan. 4, 2021 (https://www.who.int/emergencies/diseases/novel-coronavirus-2019) since the first case was reported in late December 2019 from Wuhan, China [1]. We and other groups previously reported that patients with lung, hematological, and gynecologic cancers exhibited a significant increase in mortality rate [2–10]. Accordingly, professional societies which include ASCO, IDSA, ESMO, and ACS among others, are proactively engaged in giving practical recommendations and instructions and issuing clinical guidelines, to ensure appropriate clinical management and resource allocation for cancer patients, which is critical for prevention and treatment of cancer patients during the pandemic.

So far, several countries have managed to suppress transmission by locking down schools and non-essential businesses as part of a comprehensive set of measures, while many countries are still suffering from the unprecedented pandemic and are entering second and third waves of infection. The speed and scale of COVID-19’s escape from control mechanisms have prompted urgent efforts to investigate a number of critically important issues for cancer patients: 1) Is there a difference in the immunological memory to SARS-CoV-2 between convalescent COVID-19 patients with and without cancer? 2) Dose anti-tumor treatment alter immunological memory making patients more susceptible to reinfection? These questions are of great relevance to patient management as well as to public health issues.

A previous study reporting that antibodies to SARS-CoV-2 only lasted for less than 2 months in the convalescent serum in a subset of convalescent patient caused alarm [11]. However, broad and robust memory CD4^+^ and CD8^+^ T cells persist for longer periods in convalescent patients and may mediate resistance to reinfection [12]. Indeed, the number of patients with demonstrable reinfection with SARS-CoV-2 remains very low. These results require the elucidation of the T and B cell immune response in recovery from SARS-CoV-2 and longer-term protective immunity to inform therapeutic interventions and vaccine design.

A number of cancer societies have recommend that chemotherapy should be staved off until full resolution of symptoms and viral testing becomes negative, with the potential that the risk of developing severe events could be raised by oncologic therapy, cytotoxic chemotherapy especially [13]. However, implications of these recommendations to long term outcomes from the patient’s cancer as well as a detailed understanding of the consequences on active infections remain uncertain. Firstly, considering that the current understanding of SARS-CoV-2 Virology and COVID-19 pathology are limited, the reasonable time point to resume the interrupted chemotherapy after resolution of COVID-19 infection requires further clarification. Secondly, current researches have demonstrated conflicting data concerning the association between receiving cytotoxic chemotherapy or other anti-tumor treatment and COVID-19 disease prognosis. In particular, receipt of chemotherapy [14, 15] and immune checkpoint inhibitors (ICIs) [16, 17] was associated with poor prognosis in COVID-19 patients with tumor in some studies. In contrast, other studies indicated that there is no certain correlation to draw an absolute conclusion [4, 15, 18, 19]. Intriguingly, certain anti-neoplastic hormonal therapies were reported to play a potentially protective role in SARS-CoV-2 infected population [20]. This complexity in clinical and biological reciprocity between COVID-19 and cancer and in particular cancer therapy therefore warrants further scope of the potential underlying molecular and biological mechanisms.

Herein, single-cell RNA sequencing (scRNA-seq) technology was implemented, facilitating an unbiased and relatively comprehensive visualization of the immune memory of peripheral blood mononuclear cells (PBMCs) pre- and post- anti-cancer treatment in gynecologic tumor patients recovering from COVID-19. Accordingly, we delineate the molecular characteristics of convalescent COVID-19 patients with gynecological tumor and the effect of anti-tumor therapy on their immune memory response at single cell resolution.

## RESULTS

### Single cell landscape of PBMCs of patients with gynecologic tumor and COVID-19

**Figure 1:**
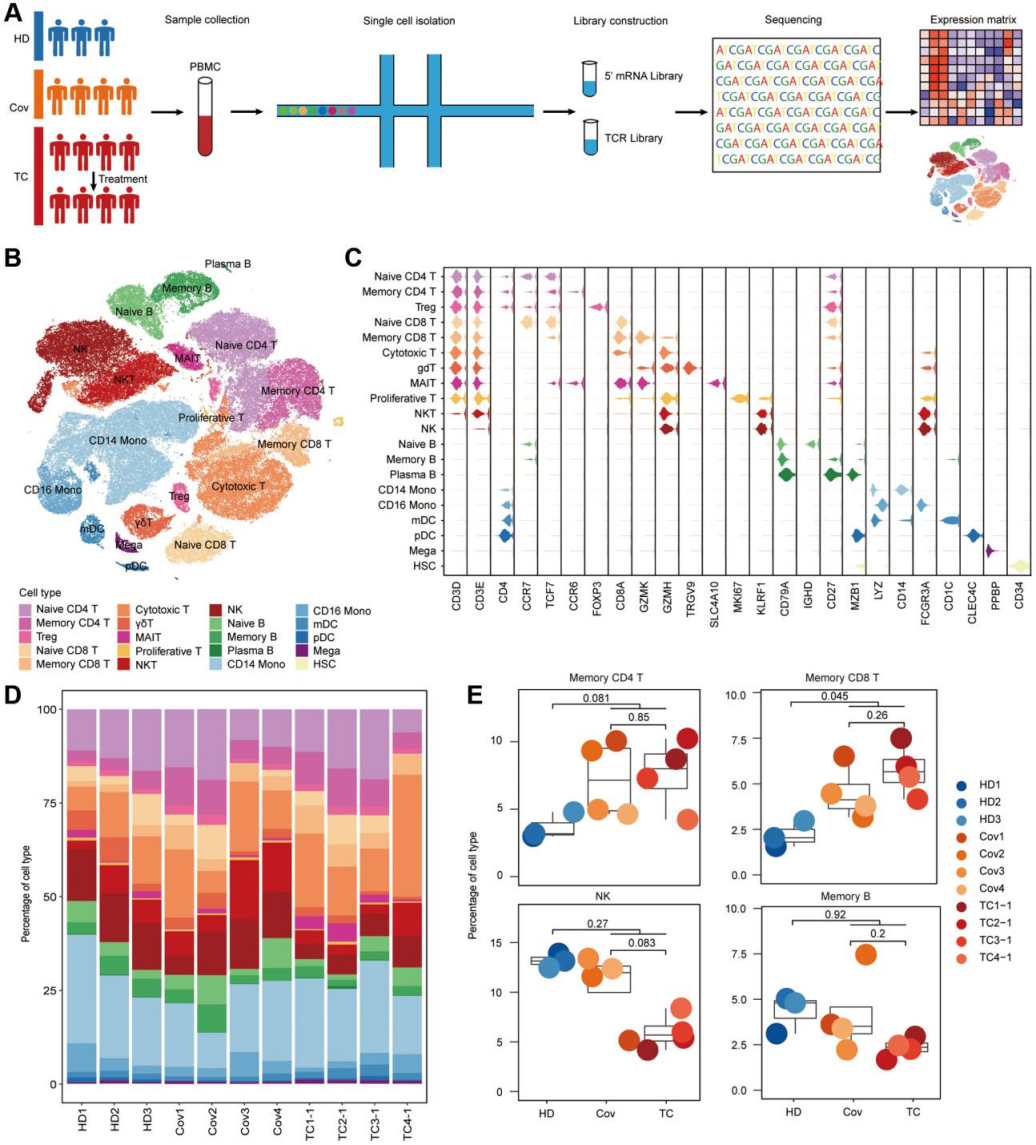
**Differences in cell compositions by single-cell transcriptomes of PBMCs.** (**A**) Schematic of the study design. (**B**) t-SNE plot of cells colored by cell types identified from HDs (*n* = 3), Covs (*n* = 4), TCs before treatment (*n* = 4). (**C**) Expression of selected canonical markers using violin plots in each cell type. (**D**) Proportion of each cell type in each sample. Bars are colored by cell types. (**E**) Box plots of proportion of cell types in each group. Shown are Memory CD4^+^ T, Memory CD8^+^ T, NK and Memory B cells. Samples are shown in different colors. Horizontal lines represent median values, with a maximum of 1.5× interquartile range. *t*-tests were conducted between each group and *p*-values indicated. *t* test was performed using R (version 4.0). ^*^p < 0.05, ^**^p < 0.01, ^***^p < 0.001.

By performing 10X Genomics scRNA-seq, we studied the transcriptomic profiles of each PBMCs from 4 gynecological tumor patients recovering from COVID-19 and undergoing cancer treatment (TCs), 4 COVID-19 convalescent (Covs) patients without cancer and 3 healthy donors (HDs) with no cancer history (Figure 1A and Supplementary Table 1). Using the unified single-cell transcriptomic analysis pipeline (see Methods), totally, 111,798 cells across all HDs, Covs and TCs, were integrated into an unbatched dataset. On average, there are 10,163 cells per sample.

Using unsupervised clustering of t-distributed stochastic neighbor embedding (t-SNE), 19 cell populations were identified by matching the expression of canonical gene markers for different cell types (Figure 1B, 1C and Supplementary Figure 1). The relative proportions of each immune cells were analyzed, revealing the discrepancies in cell composition between TCs and Covs and to compare them with that of HDs (Figure 1D, 1E). While significant differences were observed between COVID-19 patients (TCs and Covs) and HDs, indicating that SARS-CoV-2 immune memory was maintained in convalescent patients, there were limited differences between TCs and Covs. Compared to that in HDs, there is a significant increase COVID-19 patient in the proportion of memory CD8^+^ T cells (TCs and Covs), while memory CD4^+^ T cells were mildly elevated in TCs and Covs although there was no statistical difference (Figure 1E). SARS-Cov-2 specific memory T cells inducted after infection is a crucial participant in long-term protection [21]. Specific memory CD4^+^ T cells are necessary to stimulate potent B cell responses, resulting in subsequent antibody affinity maturation [22]. However, no significant difference in memory B cells was observed among three groups. This was not surprising as upon viral clearance, B cells will no longer be stimulated and proliferation is therefore terminated [23]. And a rapid decline in antibodies has been reported in mild cases [24]. Natural killer (NK) cell, which is a population of cytotoxic lymphocytes taking part in innate immunity against viral infection and tumor [25], were decreased in TCs, albeit not significantly (Figure 1E). Whether the reduction in NK cells is a risk factor for severe disease and mortality among patients with cancer and COVID-19 remains to be further determined.

### Functional changes of T cells and B cells between convalescent Covs and TCs

**Figure 2:**
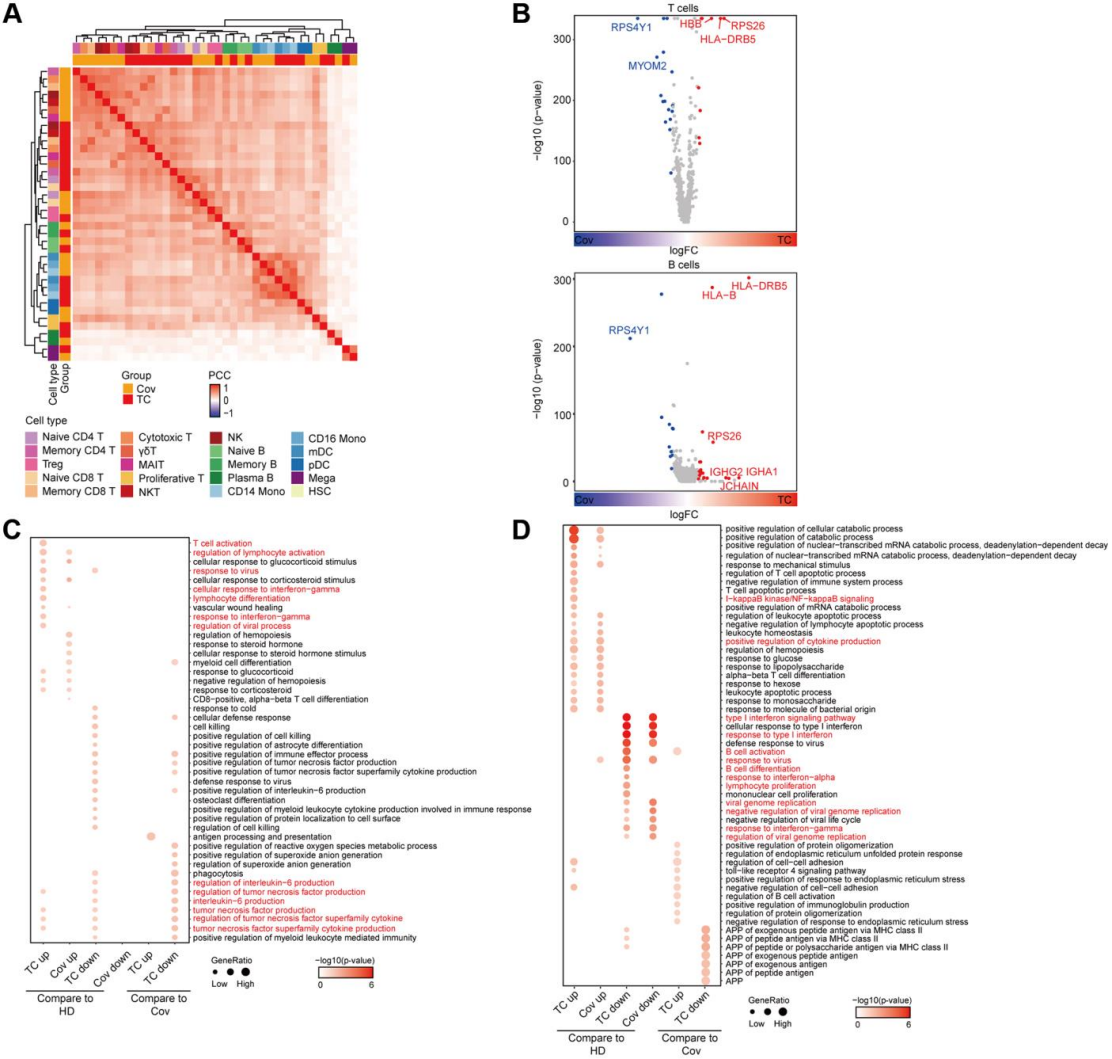
**Analysis of functional changes between TCs and Covs.** (**A**) Hierarchical clustering using the Pearson Correlation Coefficient (PCC) of a normalized transcriptome change between disease groups and HDs at cell type resolution. The color intensity indicates the PCC and the color bars above the heatmap indicate the cell type and disease group. (**B**) Differentially expressed genes in TCs compared to Covs in T and B cells. Red dots represent genes upregulated in TCs (logFC ≥ 0.25 and adjusted *p* < 0.05), while blue dots represent genes downregulated in TCs (logFC ≤ −0.25 and adjusted *p* < 0.05). Genes with |logFC| > 0.5 were labeled by gene symbols. (**C**, **D**) Enriched GO pathways of COVID-19 groups in T (**C**) and B (**D**) cells (left 4 columns: DEGs for TCs and Covs compared to HDs, right 4 columns: DEGs between TCs and Covs). The color intensity indicates the enrichment *p*-values and the point size indicates the ratio of gene enrich in each pathway.

Next, we performed hierarchical clustering to delineate the molecular differences of each cell type in Covs and TCs, according to the relative changes in gene expression compared to HDs. All T cell types and innate immune cell types, including NK, Monocyte, and DC cells clustered together based on disease group instead of by cell-types. with exceptions lied in B cells, hematopoietic stem cells (HSCs) and megakaryocytes (Figure 2A). Moreover, the difference between the two groups across cell types was limited (Figure 2A), which indicates the molecular characteristics of PBMCs in TCs are similar to that in Covs.

Further investigation of the transcriptomic changes of adaptive immune cells during recovery from SARS-CoV-2 infection was conducted by comparing the expression patterns of T and B cells in Covs or TCs with that of HDs. Consistent with previous reports [26, 27], we found T cell and lymphocyte activation, lymphocyte and myeloid cell differentiation, interferon (IFN) responses and response to virus pathways in T cell types were significantly upregulated in Covs and TCs compared to HDs (Figure 2B, up and 2C). Interestingly, in TCs IL-6 and TNF production were suppressed compared to Covs and HDs potentially due an interaction between the immune system and the tumor (Figure 2C). IL-6 can stimulate B cell proliferation, differentiation, and antibody production [28], increase antitumor activity of CD4^+^ and CD8^+^ T cells [29], and tumor necrosis factor (TNF) contributes to activation of antigen presenting cell (APC) functions, the recruitment and activation of effector CD4^+^ T cells, effector CD8^+^ T cells, and the recruitment of NK cells as well [30]. Given above, our results indicate that TCs have a unique tumor-related molecular background, which is characterized by IL6&TNF associated immune suppression.

For B cells, similar to T cells, elevated and down-regulated pathways in Covs and TCs were basically the same compared to HDs, which suggested a consistent response of adaptive immunity to SARS-CoV-2 infection between Covs and TCs (Figure 2B, bottom and 2D). Cytokine production and nuclear factor (NF)-κB signaling pathways were significantly enriched in COVID-19 patients (Covs and TCs), while defense response to virus, B cell activation and differentiation, viral genome replication, and response to IFN-γ pathways were unexpectedly reduced in COVID-19 patients (Figure 2D). In accordance with the alteration in the proportion of memory B cells in Figure 1E, the immune response of B cells to SARS-CoV-2 had likely declined in convalescent patients due to virus clearance. During the outbreak in 2003, it was reported that those infected with SARS-CoV generated durable T cell responses lasting six years. In contrast, long-term memory B cells were absent [31]. These results suggest that compared with humoral immunity, cellular immunity may play a more important role in prevention of reinfection.

### Change of immune cell composition and function after anti-tumor treatment

Long-term interruption of anti-tumor treatment risks tumor progression. In the meanwhile, patients may experience anxiety and stress during this time period. Conversely, immunosuppressive chemotherapy in convalescent COVID-19 patients risks inducing COVID-19 reactivation. To investigate the effect of recent cancer treatment, including cytotoxic chemotherapy and immunotherapy on immunity in convalescent COVID-19 patients, we performed 10X Genomics of 7 PBMCs from 3 COVID-19 convalescent patients with active gynecologic cancers pre- and post- treatment. Two of them received chemotherapy, of which one patient received a paclitaxel and cisplatin regimen (TP), another received a cisplatin, doxorubicin, and cyclophosphamide (PAC) regimen, and the other received immunotherapy (Supplementary Table 2). Cancer treatment of all three patients had been delayed due to pandemic (Supplementary Figure 2).

**Figure 3:**
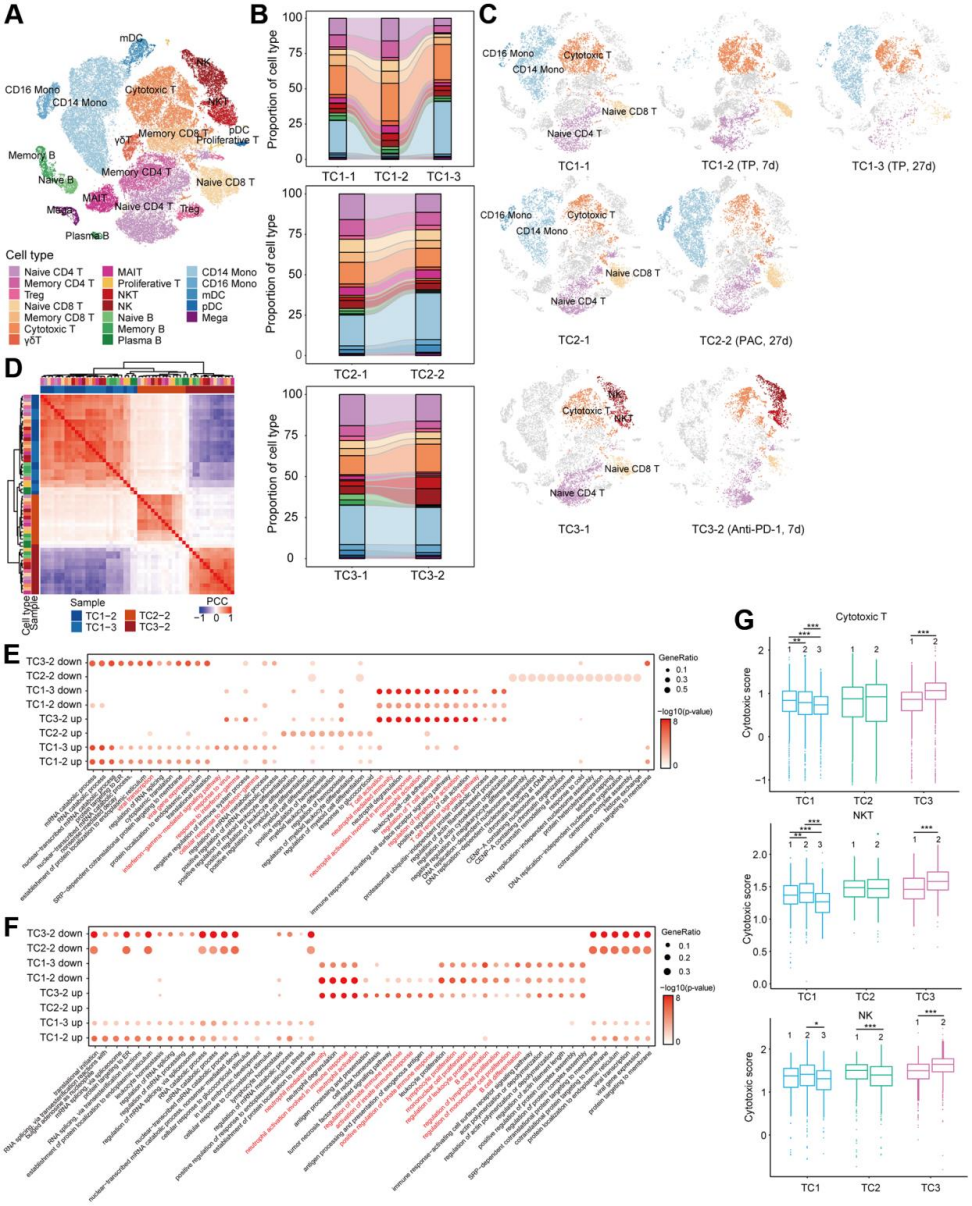
**Compositional and functional changes of cells pre- and post-treatment in COVID-19 patients with tumor.** (**A**) t-SNE plot of cells colored by cell types identified from COVID-19 patients with tumor pre- and post-treatment. (**B**) Proportion of each cell type in each person before and after treatment. Bars are colored by cell types. (**C**) t-SNE plot of cells in each person at different time point before and after treatment. (**D**) Hierarchical clustering using the Pearson Correlation Coefficient (PCC) of a normalized transcriptome change between post- and pre-treatment in lymphocytes at cell type resolution. The color intensity indicates the PCC and the color bars above the heatmap indicate the cell type and disease group. (**E**, **F**) Enriched GO pathways of COVID-19 patients with tumor after treatment in T (**E**) and B (**F**) cells (top 4 columns: downregulated GO pathways after treatment, bottom 4 columns: upregulated GO pathways after treatment). The color intensity indicates the enrichment *p*-values and the point size indicates the ratio of gene enrich in each pathway. (**G**) Box plot of cytotoxic scores for each patient in cytotoxic T, NKT and NK cells. ^*^p < 0.05, ^**^p < 0.01, ^***^p < 0.001.

19 cell populations were identified based on the expression of canonical cell-type gene markers (Figure 3A) and the relative percentage of naïve CD4^+^ and CD8^+^ T cells decreased after treatment respectively in all three patients (Figure 3B, 3C). Of note, the relative abundance of CD14^+^ and CD16^+^ monocyte decreased sharply at 7 days after TP treatment (Figure 3B, 3C, up) and were restored at Day 27 after treatment. A similar change was observed in the patient treated with PAC. Due to severe bone marrow suppression 7 days after PAC treatment, this patient’s sample failed to meet sequencing requirements (Figure 3B, 3C, medium). The two chemotherapy regimens had little effect on the relative proportion of T and B cells (Figure 3B, 3C). In conformity to a previous report which demonstrated that PD-L1 blockade induced proliferation of CD8^+^ T and NK cell subsets [32, 33], NK cells, cytotoxic T cells, and NKT cells were massively increased in the third patient at Day 27 after treatment with anti-PD-L1 (Figure 3B, 3C, bottom).

Next, to evaluate molecular changes in each cell type after treatment, we performed hierarchical clustering according to relative gene expression changes compared with the pre-treatment sample. Interestingly, for all cell types of PBMC, cells clustered based on treatment groups instead of by cell-types (Figure 3D and Supplementary Figure 3A). Obviously, molecular characteristics of PBMCS varied significantly among treatment groups, which is regardless of cell-type. Hence, we attempted to identify variations in T and B cells in relevant biological functions after treatment through gene ontology (GO) analyses. Most importantly, the enriched pathways of up-regulation and down-regulation after anti-PD-L1 treatment were exactly the opposite of that after chemotherapy in T, B and monocyte cells (Figure 3E, Supplementary Figure 3B). We observed that genes upregulated after anti-PD-L1 treatment in T cells were involved in processes including T and lymphocyte cell activation, T cell receptor signaling pathway, and neutrophil mediated immunity (Figure 3E), and in B cells were enriched in neutrophil mediated immunity, B cell activation, and lymphocyte proliferation (Figure 3F). Neutrophil mediated immunity pathway was also enriched in monocyte and dendritic cells (DC) after anti-PD-L1 treatment (Supplementary Figure 3B, 3C). Significant activation of neutrophil mediated immunity in T and B cells after anti-PD-L1 treatment suggests that neutrophils may act as APCs via direct interaction with T and B cells or exert regulatory effects on adaptive immunity [34, 35].

In accordance with the DEGs enrichment results, we found that CD8^+^ T cells, NKT cells, and NK cells all showed significantly higher cytotoxic scores after anti-PD-L1 treatment in TC3 patient, while all three subsets had lower cytotoxic scores after TP treatment in TC1 patient (Figure 3G). No obvious changes were found in TC2 patient, except that the cytotoxic score in NK cells was significantly decreased after PAC treatment (Figure 3G).

### Change of clone status of T cells after treatment in TCs

**Figure 4:**
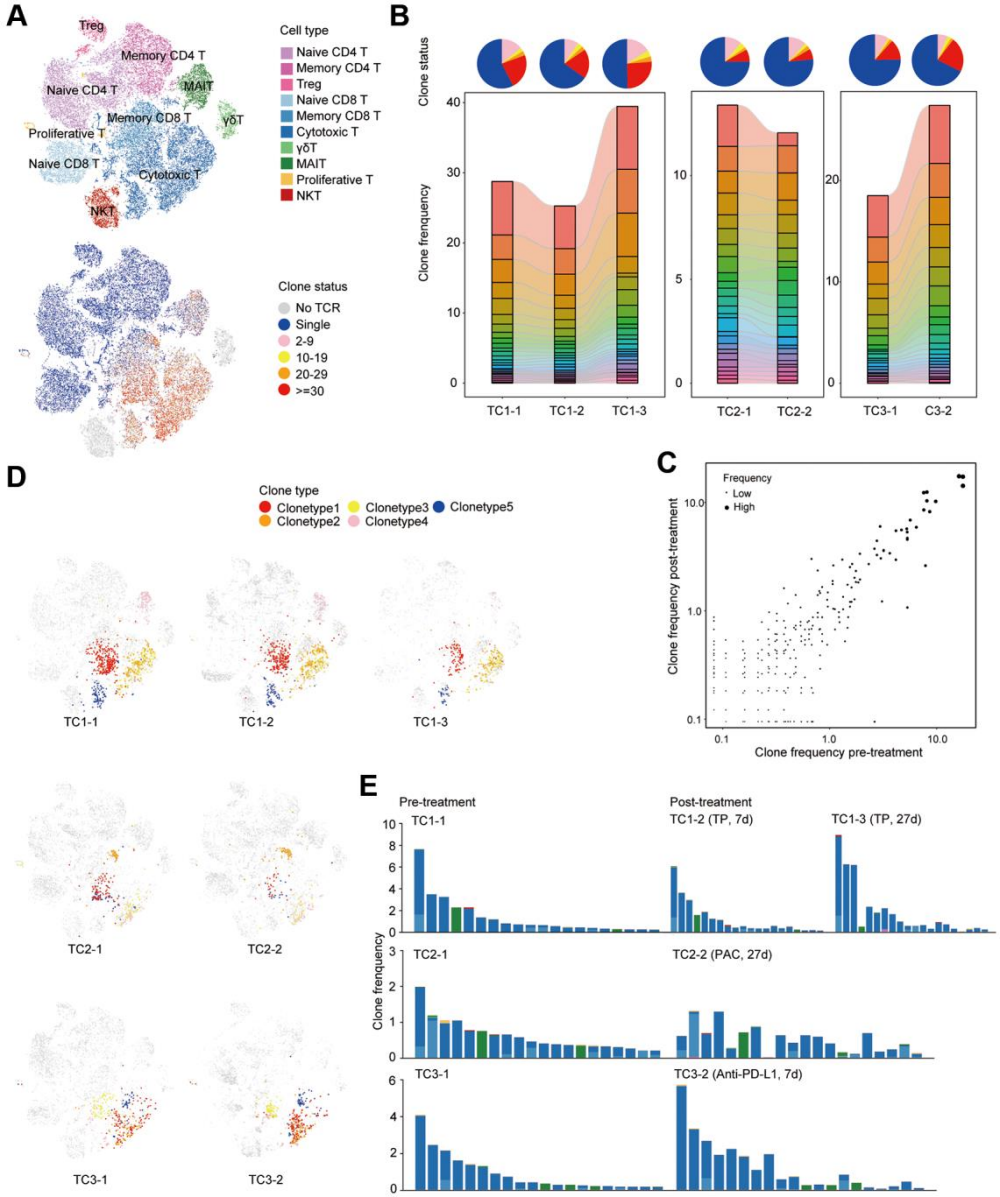
**Clonal dynamics of T cells pre- and post-treatment in COVID19 patients with tumor.** (**A**) t-SNE plot of T cells colored by cell types (top) and clone status (bottom). (**B**) The proportion of each clone status (top) and clone type (bottom). Bars are colored by the top 20 most abundant clones in each patient at different time points. (**C**) Scatterplots comparing TCR clone frequencies pre- and post-treatment. Shown are amplified clones (*n* > 1). (**D**) t-SNE plot of single cells colored by selected TCR clones. Shown are the top 5 most abundant clones before treatment in each patient at different time point. (**E**) Phenotypes of single cells belonging to the same TCR clone. Shown are the top 20 most abundant clones before treatment in each patient at different time point.

CD8^+^ T cells confront virus-infected cells with the viral antigen recognition function of T cell, which is meditated by T cell receptor (TCR). The immune response towards specific antigen is mainly determined by the diversity as well as the size of T cell receptor (TCR) repertoire [36, 37]. To gain insight into the change of clonal status of individual T cells across three conditions, TCR sequences were reconstructed based on original TCR sequencing. Briefly, most cells in all T cell subsets have matched TCR information. The exception lied in NKT and γδT cells (Figure 4A). Among T cell subsets, there are varying degrees of clonal expansion observed. Large clonal expansions with a clonal size ≥30 were mostly concentrated in cytotoxic T cells (Figure 4A), which indicated that effector T cells underwent dynamic and clonal responses in time of SARS-CoV-2 infection [26, 38].

Next, the global clone abundance was investigated based on the comparison of pre- and post- treatment frequencies of each clone in each patient (Figure 4B, 4C and Supplementary Figure 4A). We found that TP and anti-PD-L1 treatment enhanced the clonal expansion of cytotoxic T cells, while a slightly reduction was observed after PAC treatment (Figure 4B). Interestingly, most clones that were highly expanded before therapy were preserved after treatment (Figure 4C and Supplementary Figure 4A). Integration of scRNA-seq data revealed that more than 90% of highly expanded clones (*n* > 4) remained after treatment and were even maintained in a similar frequency after treatment (Figure 4D, 4E and Supplementary Figure 4B). Namely, across all patients, TOP 5 or even TOP 20 clones were still enriched in the same cell subsets (Figure 4D, 4E). Taken together, TP and anti-PD-L1 therapy had limited effects on the clonal status of T cells.

## DISCUSSION

Ongoing epidemic and potential resurgence of COVID-19 in convalescent patients is currently a major public health concern as well as a serious social issue, especially in patients with cancer, for immunosuppressing cancer treatment renders patients at a rather fragile status. In the meanwhile, immunosuppression is thought to be inherent to many types of cancer, let alone the process of cancer development itself is considered partly as a consequence of dysregulated immune surveillance [39]. Furthermore, many cancer therapies have the potential to be immunosuppressive. It still remains unclear whether tumor patients recovering from COVID-19 develop protective immunity. It was reported that specific memory T cells in convalescent patient of SARS-Cov remained to be detectable even 11 years after recovery [40]. Our results revealed that the proportions of memory CD8^+^ T cells and memory CD4^+^ T cells were increased in SARS-Cov-2 infected populations, and even higher in TCs. Consistent with this high ratio, genes related to T cell and lymphocyte activation, lymphocyte and myeloid cell differentiation, IFN responses and response to virus pathways were also highly expressed in T cell types of Covs and TCs compared to HDs. These memory T cells are likely to be critical in protecting patients against SARS-CoV-2 virus reinfection.

Recent studies suggested that antibodies may be maintained for 2-6 months after SARS-CoV-2 recovery [11, 41]. Consistent with these findings, it was reported that, in mild cases, SARS-Cov2 antibodies suffered from a rapid decline [24]. speculation aroused toward this phenomenon that ‘immunity’ to SARS-Cov-2 virus may not be long lasting. Although memory B cells may be maintained which can provide continuous protection from future infection. By rapid proliferating and differentiating into effector cells, B cells function to avoid illness progression and/or death caused by infection. In our research, the proportion of memory B cells from convalescent patients in Covs and TCs were similar to those in HDs, and defense response to virus, B cell activation and differentiation, viral genome replication, and response to IFN-γ pathways were unexpectedly reduced in COVID-19 convalescent patients. Whether these convalescent patients are able to produce a rapid humoral immune protection during future SARS-CoV-2 encounters warrants further investigation.

For physicians and researchers, one particular interest lies in the dual character of anti-tumor treatment. Whether targeted and chemo-therapies could probably potentiate SARS-CoV-2-related risks and whether this issue should give way to the risk of tumor progression. And further, whether anti-tumor treatment should be withheld, and if so, for how long. In a clinical setting, the wide range of cytotoxic agents and targeted therapies may lead to rather heterogeneous biological interactions between tumor treatment and the immune system. Our results revealed that platin-based chemotherapy mainly caused bone marrow suppression, resulting in neutropenia [42], while it had little effect on the proportion and functional phenotype of T and B cells. Moreover, TCR analysis showed that chemotherapy did not affect the clonal status of T cells, instead, TP treatment appeared to facilitate the clonal expansion of cytotoxic T cells induced by SARS-CoV-2 with a slight reduction being observed after PAC treatment. However, cytotoxic T, NKT, and NK cells all had lower cytotoxic scores after TP treatment, indicating TP may reduce cytotoxic capacity, which was not observed after PAC treatment. Taken together, clinical decision making for tumor patients during this crisis will require more robust evidence to prove the safety of specific drugs, however, contemporaneous risk assessment on a case-by-case basis is indispensable as well [43].

Immunotherapies, including ICIs, is now a part of the standard-of-care for multiple types of cancer. While leading to anti-tumor immune responses, ICIs also activate a general immune response of both innate and adaptive immune system which can result in immune-related adverse events, with pneumonitis included [44]. Though underlying lung disease is considered a risk factor of COVID-19, lung toxicity is not the most frequent adverse event of ICIs and a potential synergy between the two lung injuries is only hypothetical. In a clinical setting, ICI therapy was correlated with poor outcomes in one cohort study of COVID-19 patients [16], although the same relationship was not found in another study [18]. In our research, we found massive increase of NK cells, cytotoxic T cells, and NKT cells and these three subsets demonstrated significantly higher cytotoxic scores in patients receiving anti-PD-L1 treatment. Furthermore, anti-PD-L1 therapy could facilitate the clonal expansion of cytotoxic T cells induced by SARS-CoV-2. Possibly, these T cells could play a role in the protection against SARS-CoV-2 virus reinfection, and anti-PD-L1 could enhance the effect.

There are several limitations in our study. First, the study is limited by the small sample sizes. Thus, the difference we identified between COVID-19 patients with and without cancer require further validation by larger and methodologically robust clinical trials and/or by more retrospective studies. Second, for each cancer treatment group only one patient was assessed and it is not clear whether results in these 3 patients are sufficiently representative to contribute to clinical decision making.

Taken together, this is the first study focusing on the discrepancies of immune cell contexture between COVID-19 patients with and without gynecological malignancies pre- and post-treatment at single cell resolution. The transcriptomic data of COVID-19 patients with and without cancer at single cell level could serve as rich resource to future studies. And when integrated with corresponding TCR-based lineage information, it can facilitate better knowledge of the T cell immune response throughout the disease pathogenesis, therefore enlighten clinical interventions and vaccine design.

## CONCLUSIONS

This is the first study focusing on immune cell contexture between COVID-19 patients with and without gynecological malignancies pre- and post-treatment at single cell resolution, which may serve as a resource for deeper understanding of the T cell immune response and longer-term protective immunity to inform therapeutic interventions and vaccine design.

## MATERIALS AND METHODS

### Clinical information and data access

Approval of this study was given by the Institutional Review Board of Tongji Hospital, Tongji Medical College, Huazhong University of Science and Technology (TJ-IRB20200405). Patients were informed of necessary information in advance and an inform consent for each patient is signed. All blood samples in this study was acquired by collecting residual samples in clinical diagnostic tests, which ensured no extra burden to patients. The medical history is gained by accessing electronic medical record system which is under informed consent of patients. All data and information were used and analyzed according to ethical guidelines.

As June 7, 2020, whole blood samples of 4 COVID-19 convalescents with gynecological cancer, 4 COVID-19 convalescents without cancer and 3 healthy donors as normal controls were enrolled and 10X scRNA-seq was performed with PBMCs isolated from their blood samples. Routine laboratory tests were acquired through medical record and included in the analysis.

### 10X genomics scRNA library construction sequencing

Blood samples were collected using heparin tubes (Becton, Dickinson and Co.) for later management. For PBMC isolation and extraction, blood samples were overlaid with Ficoll-Paque Plus medium (GE Healthcare). Each heparin tube was centrifugated by density gradient, followed by Ca/Mg-free PBS resuspension and wash. The whole sample collection and processing procedure was mostly within 1h and was strictly limited to 4 h at most. The viability of PBMC cells were identified using 0.4% Trypan blue coloring. After microscope examining, samples with ≥80% viability was qualified to be incorporate into single cell library construction. In brief, in order to generate single-cell gel beads in emulsion (GEMs), the single cell suspension was loaded together with regents and Gel Beads by implementation of Chromium™ Single Cell 5’ Reagent Version 2 Kit (10x Genomics, Pleasanton, CA). Each Gel Bead contains barcoded oligonucleotides. The above operations are performed in Chromium™ Controller following manufacturer’s instructions (GemCode Technology). Inside each GEM, cells are lysed with a lysis buffer followed by barcoded reverse transcription. For each single cell, the reverse transcription of polyadenylated mRNA was distinguished by the unique barcode. Fragmented cDNA was end repaired by adding A-tailing to the 5’ end. The adaptor ligated DNA was then subjected to double sided SPRI selection. A final SPRI purification was conducted after sample index PCR. The resulting single-cell RNA-seq library was sequenced on the MGISEQ-2000 sequencer (BGI, Shenzhen, China) to insure qualification.

### Single-cell RNA-seq data processing

The single cell RNA-seq sequencing data were processed by implementing Cell Ranger (version 3.0.1, 10x Genomics) with the GRCh38 human as reference genome and the gene expression matrices were analyzed with Seurat (version 3.2.2). Cells were considered as disqualified and were removed from data processing if they met one of the following criteria: 1) UMIs below 500 or above 30,000; 2) less than 200 genes expressed; 3) more than 15% of UMIs from mitochondrial genes on the UMI counts per cell. After removal of disqualified cells, the gene expression matrix of each sample was normalized through the “NormalizeData” and “FindVariableFeatures” function with default parameters. The normalized data were then integrated using “FindIntegrationAnchors” and “IntegrateData” function with parameters set to “nfeatures = 300, dims = 1:30”. Ribosomal reads and mitochondria protein were eliminated when the integrated dataset was scaled. For PCA analysis, cells were clustered by running “FindNeighbors” and “FindClusters”, with parameter set to “k.param = 10, resolution = 1”. To visualize the clusters, data was plotted on a tSNE space, which incorporated the top 30 principle components.

### Cell type annotation

Unbiased cell type annotation was performed with the “FindAllMarker” function in Seurat. Clusters were annotated according to expressions of identified markers as well as some canonical markers of particular cell types. For better insight of the data, HSC cells and clusters which express more than 2 canonical cell-type markers were excluded from further analysis.

### Identification of differential expressed genes (DEGs) analysis and GO enrichment

Differential gene expression was carried out using “FindMarkers” function with MAST algorithm in Seurat. The false discovery rate was estimated by employing Benjamini and Hochberg procedure. Differential expressed genes (DEGs) were filtered by adopting a minimum logFC of 0.25 and a maximum FDR value of 0.05. GO enrichment of DEGs was conducted using function “enrichGO” in ClusterProfier. The parameters were set to “OrgDb = org.Hs.eg.db, ont = ’BP’, pAdjustMethod = ’BH’”.

### Hierarchical clustering of gene expression profiles at cell type resolution

Unsupervised hierarchical clustering of gene expression changes was carried out following method described previously [45]. In brief, the normalization of UMI count was conducted by multiply total UMI count of each cell type by 100,000. For each disease group, the gene expression was divided by the values in the HDs for TCs and Covs or those in the pre-treatment group respectively. Highly variable genes were determined by calculating the top 3000 standard deviation and then the data was log2-transformed. Hierarchical clustering of genes was performed according to the Pearson’s correlation coefficient (PCC) of the preciously determined highly variable genes.

### Defining cell cytotoxic scores

The cytotoxic scores were calculated according to the average expression of 12 cytotoxicity-associated genes (*GZMB*, *GZMA*, *GZMH, PRF1*, *IFNG*, *KLRK1*, *KLRB1*, *KLRD1*, *GNLY*, *NKG7*, *CTSW*, *CST7*,). The calculation was based on the function “AddModuleScore” in Seurat with default parameters.

### TCR V(D)J sequencing and data analysis

The TCR V(D)J gene segments were enriched from amplified cDNA libraries for subsequent analysis using Chromium Single-Cell V(D)J Enrichment kit (10X Genomics) following the manufacturer’s protocols. The resulting libraries were sequenced using the MGISEQ-2000 sequencer (BGI, Shenzhen, China). Demultiplex, quantification and clonotype assignment were carried out by Cell Ranger (v2.2.0) vdj pipeline with default parameters and with GRCh38 genome as reference. The frequency of each barcode was calculated and a matrix of barcode information and corresponding clonal type information was generated. Further analysis was performed on cells with following prerequisites: 1) at least one productive TCR alpha chain (TRA); 2) at least one productive TCR beta chain (TRB). A clonal type wad defined for each unique TRA(s)-TRB(s) pair.

### Statistical analysis

Statistical analysis was performed with Wilcoxon rank-sum test and *t* test using R (version 4.0) in this study, with *p*-values indicated as ^*^p < 0.05, ^**^p < 0.01, ^***^p < 0.001.

## Supplementary Materials

Supplementary Figures

Supplementary Tables
