# Supplementary material for: Impact of chemotherapy and immunotherapy on the composition and function of immune cells in COVID-19 convalescent with gynecological tumors
Source: Aging (Albany NY). 2021 Dec 4;13(23):24943–62. doi: 10.18632/aging.203739 (PMC8714165; doi:10.18632/aging.203739)
Supplement: Supplementary Figures [file aging-13-203739-s001.pdf]

SUPPLEMENTARY FIGURES

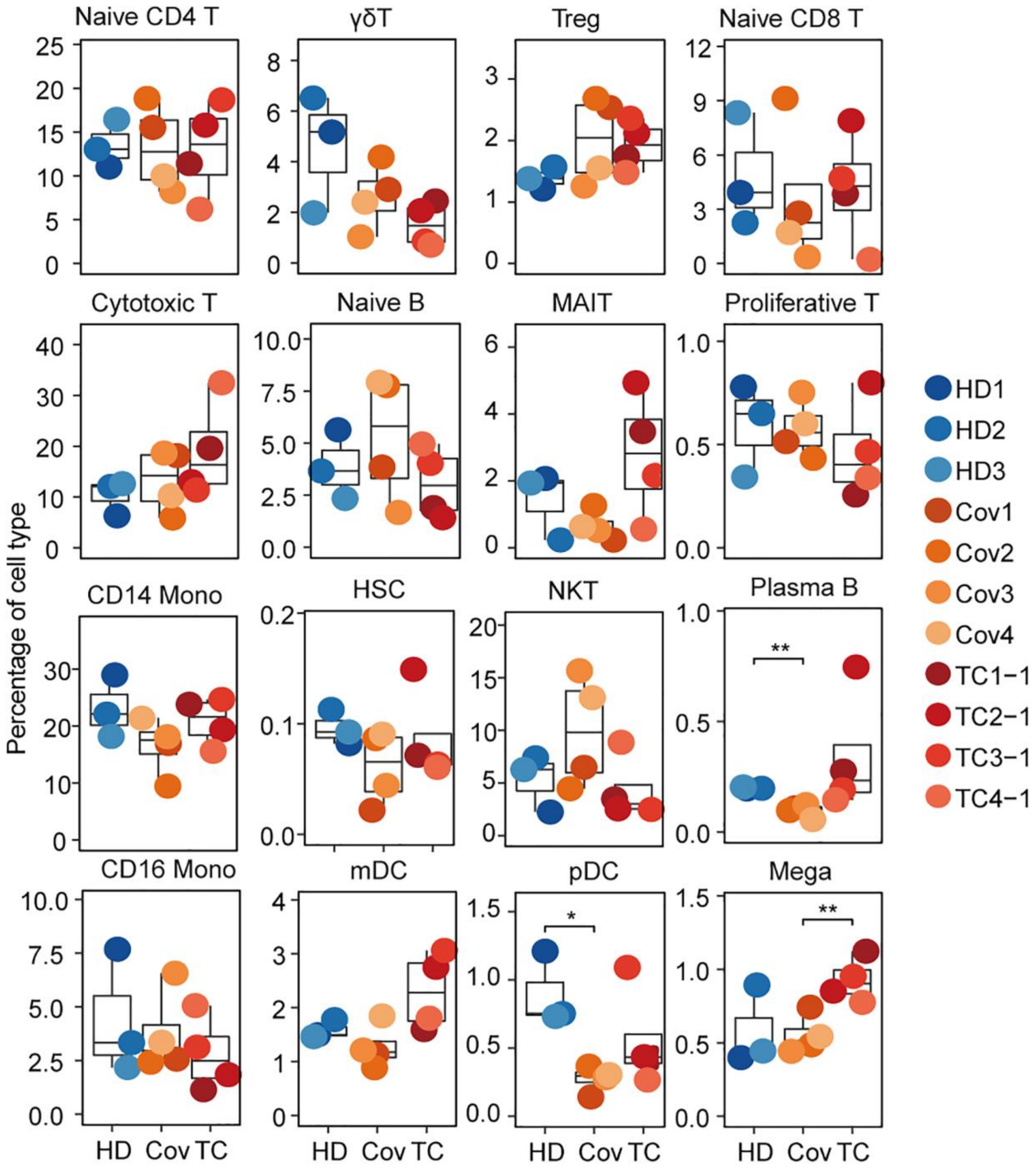

Supplementary Figure 1. Box plots of proportion of each cell type in each group. Samples are shown in different colors. Horizontal lines represent median values, with a maximum of 1.5× interquartile range. Significance was determined by Student's *t*-test. $^{*}p < 0.05$; $^{**}p < 0.01$.

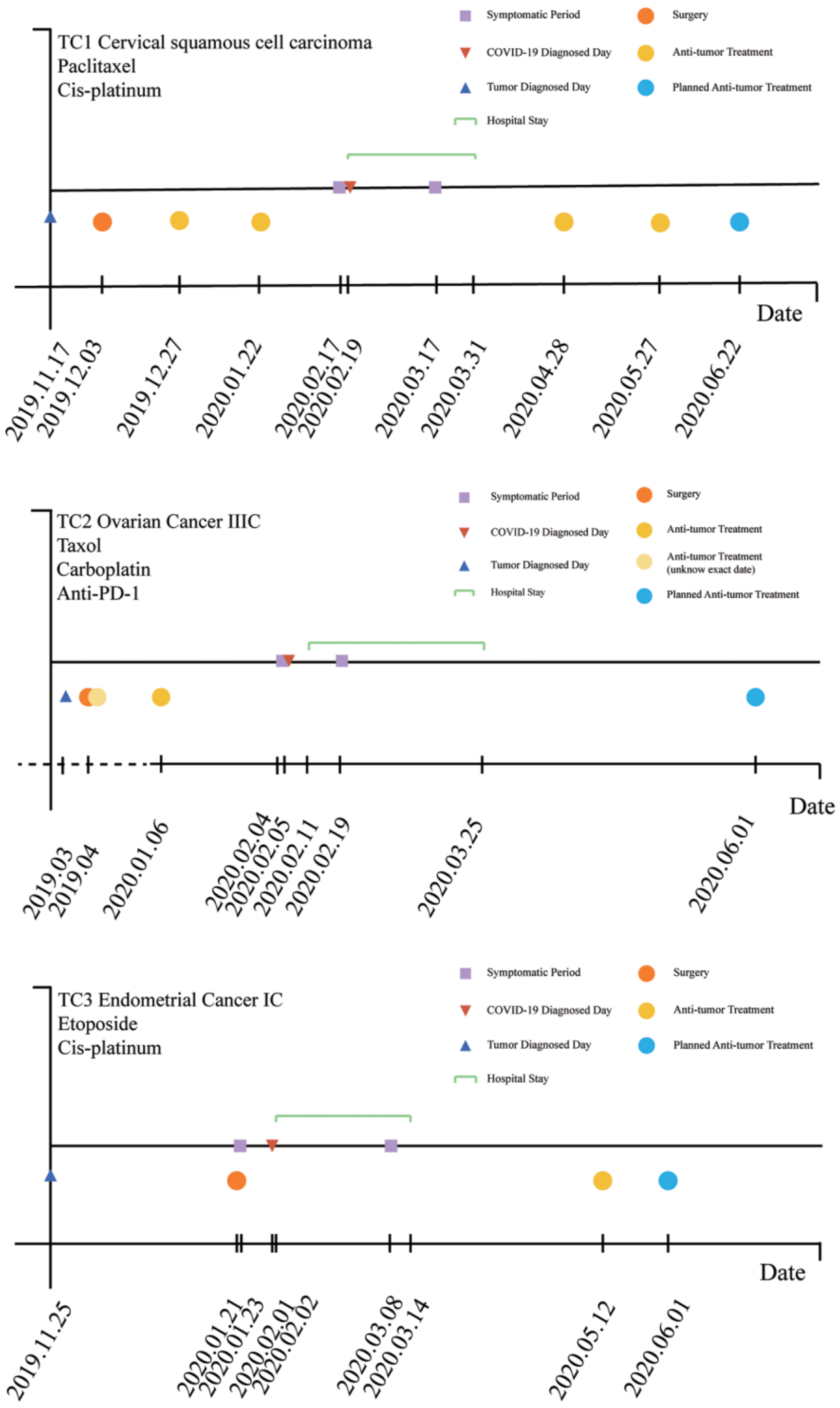

Supplementary Figure 2. Flow chart of TC patients' diagnosis and treatment during the epidemic of COVID-19. Horizontal axis represent timeline. Medical events are represented by symbols in different shape & color. Diagnose and regents are annotated above.

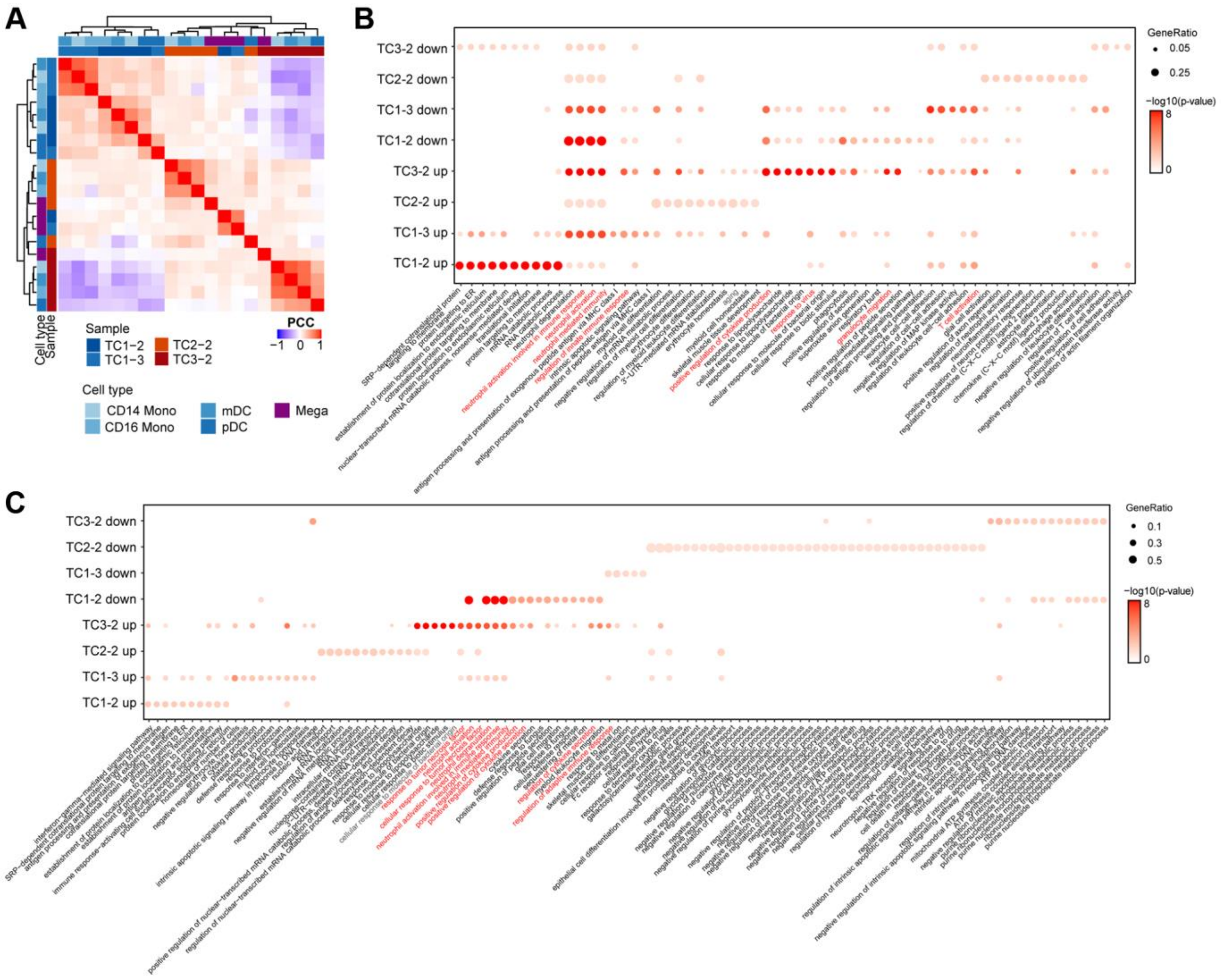

Supplementary Figure 3. Functional changes of myeloid cells pre- and post-treatment in COVID19 patients with tumor. (**A**) Hierarchical clustering using the pearson correlation coefficient (PCC) of a normalized transcriptome change between post- and pre-treatment in myeloid cells at cell type resolution. The color intensity indicates the PCC and the color bars above the heatmap indicate the cell type and disease group. (**B**, **C**) Enriched GO pathways of COVID-19 patients with tumor after treatment in Monocyte (**B**) and mDC (**C**) cells (top 4 columns: downregulated GO pathways after treatment, bottom 4 columns: upregulated GO pathways after treatment). The color intensity indicates the enrichment *p*-values and the point size indicates the ratio of gene enrich in each pathway.

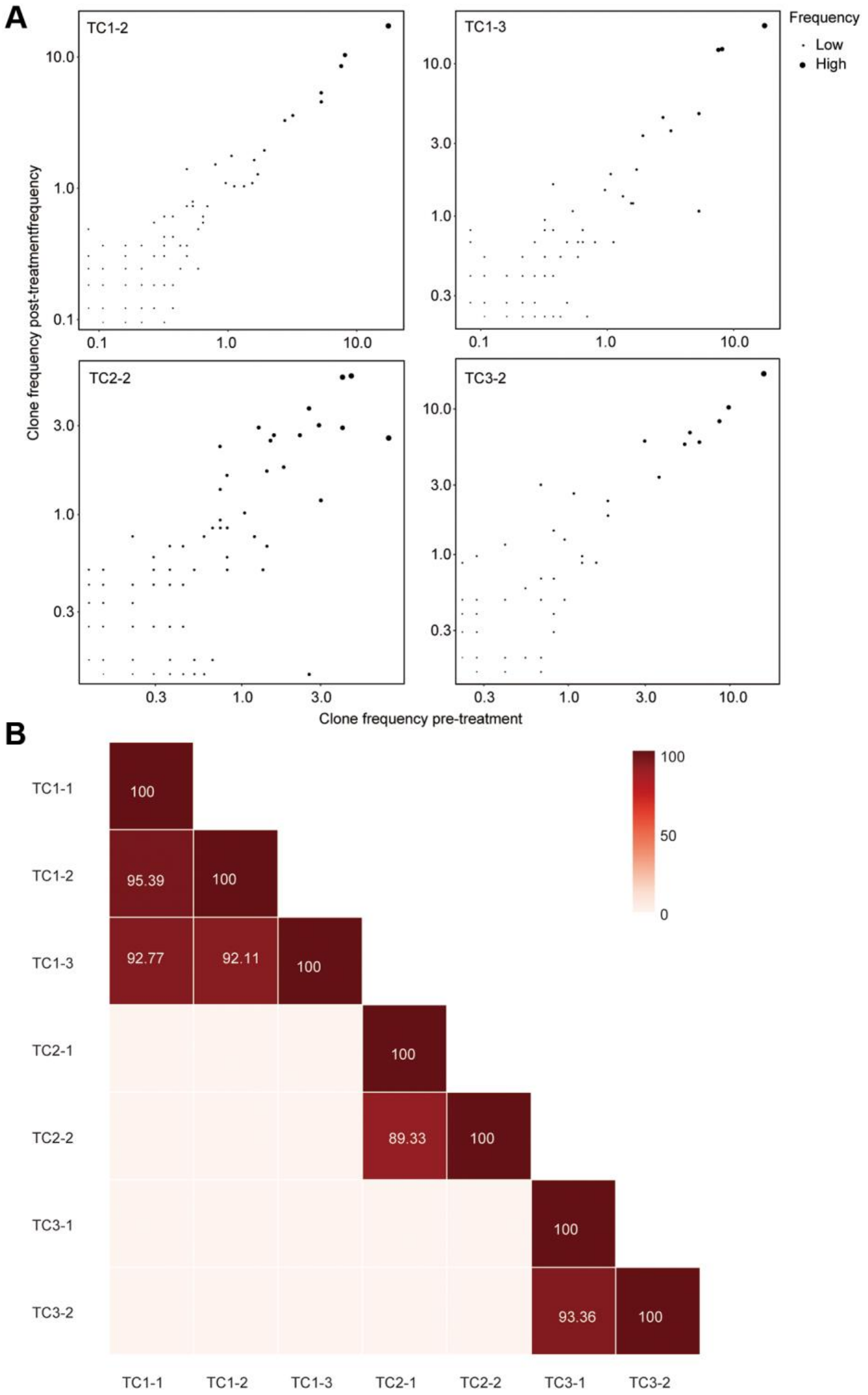

Supplementary Figure 4. Clonal dynamics of T cells pre- and post-treatment in COVID19 patients with tumor. (**A**) Scatterplots comparing TCR clone frequencies pre- and post-treatment in each sample after treatment. Shown are amplified clones (*n* > 1). (**B**) The color intensity indicates the proportion of shared clones (*n* > 4), which are labeled if they are over zero.
