# Supplementary material for: Impact of chemotherapy and immunotherapy on the composition and function of immune cells in COVID-19 convalescent with gynecological tumors
Source: Aging (Albany NY). 2021 Dec 4;13(23):24943–62. doi: 10.18632/aging.203739 (PMC8714165; doi:10.18632/aging.203739)
Supplement: Supplementary Tables [file aging-13-203739-s002.pdf]

SUPPLEMENTARY TABLES

Supplementary Table 1. Demographic characteristics and laboratory findings of enrolled patients.

	Normal Range	Covid-19 with cancer				Covid-19 without cancer				Healthy donor		
**Donor**		TC1	TC2	TC3	TC4	Cov1	Cov2	Cov3	Cov4	HD1	HD2	HD3
**Sequenced cell number**		11271	9867	9866	8586	9455	9454	11621	9144	10303	11143	11088
**Characteristics**												
Age (years)		41	26	65	60	57	59	74	59	36	43	29
Sex		F	F	F	F	F	F	F	M	F	F	F
Tumor type		Cervical squamous cell carcinoma	Ovarian hypercalcemic small cell cancer	Endometrial cancer	Endometrial cancer	NA	NA	NA	NA	NA	NA	NA
Stage		IIB	IC	IV	IIIa	NA	NA	NA	NA	NA	NA	NA
Comorbidity		NA	NA	NA	NA	NA	Otitis media	hypertension, diabetes, Thyroid nodules	NA	NA	NA	NA
Symptoms onset		cough	Fever, Cough	Fever, cough	fever, emesis, stomachache, fatigue	Fever	Fever, cough, chills, fatigue	Fever	Fever, Diarrhea	NA	NA	NA
Diagnosis		serological	PCR	PCR	PCR	CT	PCR	PCR	CT	NA	NA	NA
Severity		mild	severe	critical	severe	severe	moderate	critical	moderate	NA	NA	NA
Sample days from negative nucleic acid, days		71	62	95	55	NA	87	66	NA	NA	NA	NA
**Tumor maker**												
Neuron specific enolase, NSE, μg/L	<16.3	NA	7.92	NA	NA	NA	NA	NA	NA	NA	NA	NA
Alpha-fetoprotein (AFP), ng/ml	≤7.0	NA	2.29	3.89	4.03	NA	NA	NA	NA	NA	NA	NA
Carcinoembryonic antigen (CEA), ng/ml	≤5.0	NA	NA	5.87	0.717	NA	NA	NA	NA	NA	NA	NA
squamous cell carcinoma associated antigen (SCC), ng/ml	≤1.5	NA	NA	2.2	NA	NA	NA	NA	NA	NA	NA	NA
Carbohydrate antigen 199 (CA199), U/ml	≤34	NA	NA	13.73	10.09	NA	NA	NA	NA	NA	NA	NA
Carbohydrate antigen 125 (CA125), U/ml	≤35	NA	NA	NA	19.5	NA	NA	NA	NA	NA	NA	NA
Carbohydrate antigen 153 (CA153), U/ml	≤31.3	NA	NA	22.5	NA	NA	NA	NA	NA	NA	NA	NA
Human epididymis protein 4 (HE4), pmol/ml	≤140	NA	NA	97.3	71.3	NA	NA	NA	NA	NA	NA	NA
**Inflammation factors**												
IL-6		NA	12.87	12.93	<1.5	<1.5	<1.5	5.56	17.12	<1.5	<1.5	<1.5
TNF-α		6.2	<4	<4	12.1	NA	23.5	4.9	10.8	<4	<4	<4

Supplementary Table 2. Laboratory findings of COVID-19 patients receiving anti-tumor treatment.

	Normal Range	TC1			TC2		TC3	
**Donor**		TC1-1	TC1-2	TC1-3	TC2-1	TC2-2	TC3-1	TC3-2
**Sequenced cell number**		11271	9537	6505	9867	12051	9866	8130
**Blood routine**								
leukocyte, × 10^9/L	3.50–9.50	7.59	7.17	6.62	8.95	4.95	6.52	5.66
neutrophil, %	40.0–75.0	72.5	54.5	60.1	69.3	72.1	76.9	76.5
neutrophil, × 10^9/L	1.80–6.30	5.5	3.91	3.98	6.2	3.57	5.02	4.33
lymphocyte, %	20.0–50.0	19.2	36.7	32.5	18.4	16.4	18.7	14.8
lymphocyte, × 10^9/L	1.10–3.20	1.46	2.63	2.15	1.65	0.81	1.22	0.84
monocyte, %	3.0–10.0	7.5	7	6.6	11.3	10.7	4	7.6
monocyte, × 10^9/L	0.10–0.60	0.5	0.5	0.44	1.01	0.53	0.26	0.43
eosinophil, %	0.4–8.0	0.7	1.5	0.6	0.9	0.4	0.2	0.9
eosinophil, × 10^9/L	0.02–0.52	0.05	0.11	0.04	0.08	0.02	0.01	0.05
basophilic granulocyte, %	0.0–1.0	0.1	0.3	0.2	0.1	0.4	0.2	0.2
basophilic granulocyte, × 10^9/L	0.00–0.10	0.01	0.02	0.01	0.01	0.02	0.01	0.01
PLT, × 10^9/L	125.0–350.0	343	265	228	384	431	157	142
**Blood biochemistry**								
ALT, U/L	≤33	8	8	9	11	15	7	9
AST, U/L	≤32	14	17	14	16	14	14	13
CR, umol/L	45–84	54	43	48	89	70	58	60
BUN, mmol/L	2.6–7.5	3.4	1.6	4.5	3.6	4.2	6.04	6.4
**Tumor maker**								
Alpha-fetoprotein (AFP), ng/ml	≤7.0	NA	NA	NA	NA	1.85	NA	NA
Carcinoembryonic antigen (CEA), ng/ml	≤5.0	NA	NA	NA	NA	2.57	NA	NA
squamous cell carcinoma associated antigen (SCC), ng/ml	≤1.5	0.4	NA	0.6	NA	0.91	NA	NA
Carbohydrate antigen 199 (CA199), U/ml	≤34	NA	NA	NA	NA	28.34	NA	NA
Carbohydrate antigen 125 (CA125), U/ml	≤35	NA	NA	NA	9.5	7.59	8.4	NA
Human epididymis protein 4 (HE4), pmol/ml	≤140	NA	NA	NA	46.9	110.9	NA	NA
Serum antibody to SARS-CoV-2								
IgG, U/ml	<1.0	17.5	NA	5.5	9.79	NA	19.5	NA
IgM, U/ml	<1.0	0.62	NA	0.7	NA	NA	0.39	NA
